# Unlocking New Therapeutic Options for Vincristine-Induced Neuropathic Pain: The Impact of Preclinical Research

**DOI:** 10.3390/life14111500

**Published:** 2024-11-17

**Authors:** Ciprian Pușcașu, Simona Negreș, Cristina Elena Zbârcea, Cornel Chiriță

**Affiliations:** Faculty of Pharmacy, “Carol Davila” University of Medicine and Pharmacy, Traian Vuia 6, 020956 Bucharest, Romania; ciprian.puscasu@umfcd.ro (C.P.); simona.negres@umfcd.ro (S.N.); cornel.chirita@umfcd.ro (C.C.)

**Keywords:** vincristine, neuropathic pain, natural compounds, antioxidants, dexmedetomidine, tropisetron, aripiprazole

## Abstract

Vincristine, a vinca alkaloid, is used in chemotherapy protocols for cancers such as acute leukemia, Hodgkin’s disease, neuroblastoma, cervical carcinoma, lymphomas, breast cancer, and melanoma. Among the common adverse effects of vincristine is peripheral neuropathy, with most patients receiving a cumulative dose over 4 mg/m^2^ who develop varying degrees of sensory neuropathy. The onset of vincristine-induced peripheral neuropathy can greatly affect patients’ quality of life, often requiring dose adjustments or the discontinuation of treatment. Moreover, managing vincristine-induced peripheral neuropathy is challenging, with few effective therapeutic strategies available. In the past decade, preclinical studies have explored diverse substances aimed at preventing or alleviating VIPN. Our review consolidates these findings, focusing on the analgesic efficacy and potential mechanisms of various agents, including pharmaceutical drugs, natural compounds, and antioxidants, that show promise in reducing neuropathic pain and protecting neural integrity in preclinical models. Key novel therapeutic options, such as metabolic agents (liraglutide), enzyme inhibitors (ulinastatin), antipsychotics (aripiprazole), interleukin-1 receptor antagonists (anakinra), hormones (oxytocin), and antioxidants (thioctic acid), are highlighted for their neuroprotective, anti-inflammatory, and antioxidant effects. Through this synthesis, we aim to enhance the current understanding of VIPN management by identifying pharmacological strategies that target critical molecular pathways, laying the groundwork for future clinical studies. By clarifying these novel pharmacological approaches and elucidating their mechanisms of action, this review provides a foundation for developing more effective VIPN treatment strategies to ultimately improve patient outcomes.

## 1. Introduction

Cancer is a leading cause of morbidity and mortality worldwide, significantly impacting the quality of life of millions of patients [1]. According to the World Health Organization (WHO), an estimated 20 million new cancer cases were reported in 2022, with 9.7 million resulting in death, and approximately 1 in 5 people will develop cancer during their lifetime [2]. Chemotherapy is one of the methods used to treat cancer patients, alongside radiotherapy, surgery, and supportive treatments [3]. However, its therapeutic benefits are frequently compromised by severe adverse effects, including chemotherapy-induced neuropathic pain (CINP). CINP results from damage to the somatosensory nervous system caused by various chemotherapeutic agents and is a major cause of neuropathic pain in cancer patients [4]. This condition can significantly influence treatment decisions, such as dosage adjustments or discontinuation of therapy, thereby limiting the overall efficacy of cancer treatment [5]. There are versatile substances such as cannabidiol in terms of the pathologies it can treat, which can be administered as an adjuvant treatment in neuropathic pain or cancer [6]. Additionally, CINP adversely affects patients’ quality of life due to incomplete recovery and the financial burden associated with both acute and chronic forms of the condition [7]. Sensory symptoms usually manifest as spontaneous or induced abnormal sensations such as paresthesia, dysesthesia, numbness, burning sensations, electric shock, as well as allodynia or hyperalgesia caused by mechanical or thermal stimuli [4]. CINP may initially present as an acute pain syndrome, with sensory symptoms appearing during or immediately after drug administration, and can develop into chronic neuropathy following repeated treatment cycles [4,8]. Notably, 47% of patients continue to experience peripheral neuropathy symptoms even six years after completing chemotherapy [9].

Managing CINP remains challenging, with limited effective treatment options. ASCO (American Society of Clinical Oncology) guidelines recommend duloxetine (30–60 mg daily) based on positive trial results [10,11,12,13]. Tricyclic antidepressants, such as amitriptyline and nortriptyline, have not shown benefits for patients with CIPN and should be avoided in older adults due to risks of sedation and anticholinergic side effects [14,15]. Anticonvulsants, such as gabapentin and pregabalin, are commonly used but require careful dosing in older patients [16,17]. Topical treatments and local therapies, such as lidocaine and capsaicin, can help with mild symptoms [17,18]. Opioids are reserved for severe cases when other treatments fail, with palliative care involvement recommended [17].

Vincristine, a vinca alkaloid, is a crucial chemotherapy drug used in protocols for various cancers, including acute leukemia, Hodgkin’s disease, neuroblastoma, cervical carcinoma, lymphomas, breast cancer, and melanoma [19]. Unfortunately, vincristine-induced peripheral neuropathy (VIPN) is a common and debilitating side effect. Most patients receiving cumulative doses over 4 mg/m^2^ develop some degree of peripheral neuropathy [20,21]. The reported prevalence of VIPN ranges from 10% to 90% in pediatric patients and can be as high as 78% in adults [22,23].

In the last decade, an increasing number of preclinical studies have proven the efficacy of various substances in preventing or reducing VIPN. Considering that at present, the treatment of peripheral neuropathy remains often unsatisfactory, as many patients do not experience significant improvement with medication [24] and the high prevalence of VIPN in the population, our main purpose was to review the current status of preclinical studies assessing the analgesic efficacy of such agents, highlighting their potential underlying mechanisms. We hope to provide new pharmacological options and future perspectives for the therapeutic approach of VIPN.

## 2. Materials and Methods

A survey of the literature was conducted using PUBMED to identify the most pertinent articles on in vivo preclinical findings regarding the effects of various substances on VIPN. The search was confined to English-language publications, emphasizing recent works from 2014 to 2024 (73% of the nonclinical studies) while also considering significant older studies. The following keywords and MeSH terms were utilized: “VIPN” OR “vincristine” AND “neuropathic pain,” “neuropathy,” “neurotoxicity,” and “peripheral neuropathy”. Selection criteria for inclusion were designed to enhance study quality and relevance. Studies needed to (1) be original articles published in peer-reviewed journals, (2) involve rodent models of VIPN (specifically rats or mice), and (3) report significant findings on therapeutic agents, whether used in single therapy or in combination. Details on drug names, dosages, mechanisms of action, and the species used were required for inclusion. After a thorough eligibility assessment and cross-referencing, the final selection included studies that best aligned with these criteria for the review.

## 3. Results

In PubMed, a total of 63 articles were selected after analysis, reporting substances that mitigated VIPN in animal models. These substances can be broadly categorized into three main classes: pharmaceutical drugs, natural extracts and compounds, and vitamins and antioxidants.

### 3.1. Pharmaceutical Drugs

#### 3.1.1. Antiepileptics

Ethosuximide, lacosamide, topiramate, gabapentin, zonisamide, and pregabalin have all shown positive results in animal models of VIPN. Flatters et al. [25] found that ethosuximide, a T-type calcium current inhibitor [26] administered intraperitoneally (i.p.) at 300 mg/kg, almost completely reversed mechanical allodynia and hyperalgesia in rats, with consistent effects over three days of repeated dosing without developing tolerance. The blockade of T-type calcium channels by ethosuximide likely reduces the exocytosis of excitatory neurotransmitters by preventing the depolarization necessary to activate high-voltage-activated Ca^2^⁺ channels. Alternatively, ethosuximide may directly affect neurons early in polysynaptic pathways. Postsynaptic NMDA (N-methyl-D-aspartate) receptor-mediated post-discharge and excessive spike activity, which indicate central sensitization and neuronal hyperexcitability, appear to be particularly sensitive to ethosuximide’s effects [27].

Beyreuther et al. found that lacosamide significantly reduced thermal allodynia on a cold plate set to 4 °C when administered at doses of 10 and 30 mg/kg. Additionally, at a lower dose of 3 mg/kg, lacosamide also lessened responses on warmer surfaces, including a 38 °C warm plate and a 52 °C hot plate. Moreover, lacosamide effectively inhibited both tactile allodynia and mechanical hyperalgesia at 10 and 30 mg/kg doses [28]. Lacosamide is an antiepileptic medication that acts by blocking sodium channels. It promotes both slow and, in some instances, fast inactivation of voltage-gated sodium channels (VGSC) and interacts with collapsin response mediator protein 2 (CRMP2), which may contribute to its role in modulating pain [29,30].

Jakhar et al. [31] showed that gabapentin, topiramate, and zonisamide, when administered after a 14-day vincristine regimen, produced significant antihyperalgesic effects in the hot plate test, although these required repeated dosing for full efficacy. Topiramate is classified as a broad-spectrum second-generation antiepileptic drug [32] That possesses several pharmacological properties that contribute to its effectiveness in treating epilepsy and neuropathic pain, including modulation of voltage-gated sodium and calcium ion channels, enhancement of gamma-aminobutyric acid (GABA) inhibition, and inhibition of excitatory glutamate neurotransmission [31]. Gabapentin also acts through several mechanisms, including calcium influx blocking, reducing neuronal excitability, and glutamate release inhibition [33]. Gabapentin has demonstrated its antihyperalgesic effect in different animal models of neuropathic pain [34,35,36], being currently commonly used for CIPN management [17]. Zonisamide, a benzisoxazole analog, acts through multiple mechanisms to treat epilepsy. It inhibits voltage-gated sodium and T-type calcium channels, crucial for controlling seizures, and may also reduce glutamate release and enhance GABA function [37]. Its ability to alleviate hyperalgesia is primarily attributed to its actions in stabilizing cell membranes and modulating neuronal activity [38].

More recently, pregabalin was tested both alone and in combination with melatonin. Pregabalin alone reduced thermal hyperalgesia and improved biochemical markers such as AST (aspartate aminotransferase) and MDA (malondialdehyde). Co-administration of melatonin and pregabalin exhibited protective and analgesic effects in the context of vincristine treatment. The analgesic effect was evidenced by the tail immersion behavioral test, while histopathological investigations revealed notable protection of the sciatic nerve, indicated by reduced degeneration levels. Moreover, the combination efficiently reduced VIPN through its antioxidant capacity [39]. Pregabalin works by modulating excitatory neurotransmission through α2-δ subunits on voltage-gated calcium channels, leading to reduced neurotransmitter release [40].

#### 3.1.2. Antidepressants

The serotonin-norepinephrine reuptake inhibitors (SNRIs) milnacipran and duloxetine showed promising results in preclinical VIPN models. Milnacipran exhibits analgesic effects across various pain models. By blocking the reuptake of serotonin and norepinephrine, milnacipran effectively alleviates chronic pain conditions like fibromyalgia through modulation of neurotransmitter levels in inhibitory pathways of the central nervous system [41]. Katsuyama et al. examined the effects of various doses of milnacipran (10, 20, and 40 mg/kg i.p.) in an animal model of VIPN. A single dose of milnacipran (40 mg/kg, i.p.) did not alleviate vincristine-induced mechanical allodynia. However, repeated daily administration of milnacipran (20 or 40 mg/kg, i.p.) over a period of 7 days resulted in a significant reduction in vincristine-induced mechanical allodynia. [42].

Similarly, duloxetine, administered at daily doses of 5–20 mg/kg over seven days, significantly decreased pain sensitivity in a mouse model [42]. In a separate study, duloxetine at higher doses (10, 20, and 30 mg/kg/day) for six weeks improved nerve conduction velocity and reduced microglial activation, suggesting that extended treatment may yield greater benefits [43]. These findings align with clinical evidence, making duloxetine the recommended first-line treatment for CIPN in cancer patients, as per ASCO’s guidelines [10].

#### 3.1.3. NMDAR (N-Methyl-D-Aspartate Receptor) Antagonists

Amantadine and memantine, both NMDAR antagonists, demonstrated potential in mitigating VIPN. Amantadine was tested at oral doses of 2, 5, 12, 25, and 50 mg/kg daily for 14 days. The higher doses (25 and 50 mg/kg) significantly reduced mechanical hyperalgesia in rats, showing a dose-dependent effect. It also activated anti-inflammatory cytokines, lowered pro-inflammatory markers, and increased antioxidant enzyme levels such as SOD (superoxide dismutase) and CAT (catalase), highlighting its neuroprotective properties [3]. Amantadine, traditionally used as an antiviral for influenza and in the treatment of Parkinson’s disease, is also being explored for pain management. This interest stems from its ability to act as a non-competitive antagonist of NMDAR, which plays a role in the neurophysiological processes of nociception [3]. Recent research highlighted that amantadine reduced hypersensitivity and oxidative stress while increasing GSH (glutathione) levels in a rat model of neuropathic pain [44]. Moreover, Dogan et al. found that amantadine decreased TNF-α (tumor necrosis factor α), MDA, and MPO (myeloperoxidase) levels, promoted angiogenesis, and modulated inflammation and apoptosis [45].

Memantine, a derivative of amantadine, was tested at 2.5, 5, and 10 mg/kg in rats over 12 days. Only the highest dose (10 mg/kg) increased the paw withdrawal threshold, indicating its potential to alleviate VIPN at higher concentrations [46]. Memantine is clinically used for managing Parkinson’s disease, spasticity, and Alzheimer’s disease, making it a candidate for repurposing in VIPN [47,48]. Memantine acts as a noncompetitive antagonist of NMDAR, distinguished by its superior patient tolerance due to its rapid off-rate as an open-channel blocker compared to ketamine. In the spinal nerve ligation model, memantine has demonstrated greater effectiveness in alleviating allodynia with minimal motor impairment compared to ketamine. Moreover, memantine showed efficacy in nonclinical studies of different pain models by reversing mechanical sensitivity and reducing proinflammatory cytokine levels.

#### 3.1.4. Metabolic Agents

Simvastatin, a statin that inhibits the enzyme HMG-CoA reductase, is used to treat cardiovascular diseases such as atherosclerosis, stroke, and peripheral arterial disease [49], and liraglutide, a long-acting GLP-1 (glucagon-like Peptide-1) analog that enhances insulin secretion from pancreatic β cells [50], have both been investigated for their potential to alleviate VIPN in rodent models.

Simvastatin, administered at 7.5, 15, and 30 mg/kg over two weeks, significantly reduced neuropathic pain at lower doses without affecting cholesterol levels. However, higher doses (30 mg/kg) induced neuropathic pain in normal rats, indicating a narrow therapeutic window [49]. Statins are widely prescribed for managing cardiovascular conditions like atherosclerosis, stroke, and peripheral arterial disease, primarily due to their cholesterol-lowering properties. Additionally, they exhibit various cholesterol-independent effects, including antioxidant, anti-inflammatory, antimicrobial, neurotrophic modulation, and upregulation of L-type Ca^2+^ channels, which affect smooth muscle contraction [49]. Simvastatin effectively suppressed nerve ligation-induced interleukin-1β expression in the sciatic nerve and mitigated the activation of spinal microglia and astrocytes following sciatic nerve injury [51]. Furthermore, another study proposed that simvastatin’s ability to relieve chronic neuropathic pain may stem from its inhibition of actin-mediated intracellular trafficking through the Rho/LIMK (LIM kinase)/cofilin pathway. Rho activation triggers inflammatory cytokine activity, contributing to pain development. Within this framework, Rho serves as a critical link connecting statins to their anti-inflammatory and immunomodulatory effects [52].

Liraglutide (1 mg/kg) was tested in a VIPN model for five weeks. Liraglutide mitigated electrophysiological impairments, reduced lipid peroxidation, and preserved nerve growth factor (NGF) expression, suggesting a protective role against VIPN [53]. Previous studies have demonstrated the cytoprotective effects of GLP-1 analogs [54,55]. Research also suggests that GLP-1 analogs may offer therapeutic benefits for central and peripheral degenerative changes observed in animal models of Alzheimer’s disease and diabetes [56,57]. Moreover, recent findings show that pretreatment with liraglutide protects neuronal cells from oxidative stress and glutamate-induced excitotoxicity [55].

#### 3.1.5. Enzyme Inhibitors

Enzyme inhibitors such as cilostazol, propentofylline, and ulinastatin showed potential in reducing VIPN symptoms through anti-inflammatory and neuroprotective mechanisms. Cilostazol, a selective phosphodiesterase (PDE)-3 inhibitor used to treat various vascular dysfunctions [58], was investigated for its effects on VIPN. Thacheril Mohanan et al. demonstrated that a 5-day treatment with cilostazol at doses of 20 and 40 mg/kg significantly reduced both mechanical hyperalgesia and allodynia in the VIPN model [59]. Koyanagi et al. reported that cilostazol may prevent Schwann cell dedifferentiation by increasing cyclic AMP (adenosine monophosphate) signaling through the inhibition of phosphodiesterase (PDE), indicating its potential to alleviate chemotherapy-induced peripheral neuropathy [60].

Similarly, propentofylline, a nonselective PDE inhibitor, alleviated mechanical allodynia and reduced microglial and astrocytic activation in the spinal cord after daily administration of 10 mg/kg [61]. In addition to its PDE inhibitory activity, it also inhibits adenosine reuptake and acetylcholinesterase [62]. It offers neuroprotective benefits by suppressing glial cell activation, which in turn lowers the levels of pro-inflammatory cytokines, free radicals, and prostaglandins [63].

Ulinastatin inhibits tryptase and other proteolytic enzymes, including trypsin, neutrophil elastase, other serine proteases, hyaluronidase, and cathepsin G, reduces inflammation, scavenges free radicals, and protects organ function. It is widely used to treat pancreatitis, shock, sepsis, and inflammatory diseases and is beneficial in cancer and surgical patients for its anti-inflammatory effects and ability to activate urokinase-type plasminogen [64,65,66]. Recent studies show that ulinastatin effectively alleviates neuropathic pain in rat models with spinal nerve injuries by suppressing proinflammatory cytokines like TNF-α and IL-6 while simultaneously enhancing levels of the anti-inflammatory cytokine IL-10 [67,68,69]. Nie et al. investigated the efficacy of ulinastatin (0.5–5.0 × 10^5^ U/kg), both alone and in combination with dexmedetomidine, an α2-adrenergic agonist, for reducing VIPN. Their study demonstrated that ulinastatin alone significantly reduced vincristine-induced mechanical allodynia in a dose-dependent manner and restored interleukin-10 (IL-10) levels with an ED_50_ value of 4.20 × 10^5^ U/kg. The combination of ulinastatin and dexmedetomidine further enhanced these effects, suggesting a synergistic interaction [64]. This synergy is likely due to the combined action of IL-10 and the α-2 adrenergic receptor in the dorsal root ganglion, with IL-10 acting as a potent anti-inflammatory cytokine that suppresses proinflammatory responses, reduces nociceptor sensitization and provides analgesia [70,71]. Additionally, α-2 adrenergic receptors have been associated with increased IL-10 production under subchronic stress [72]. Notably, the combined administration of ulinastatin and dexmedetomidine increased IL-10 expression and inhibited vincristine-induced mechanical allodynia more effectively than either agent alone. This synergistic effect was reversed by the α-2 adrenergic receptor antagonist yohimbine and IL-10 siRNA (small interfering RNA), suggesting that the α-2 adrenergic receptor may be the upstream regulator of IL-10 expression. These findings imply that the α-2 adrenergic receptor and IL-10 may constitute a shared pathway for achieving synergistic analgesic effects when using ulinastatin and dexmedetomidine [64]. 

#### 3.1.6. Alpha-2 Adrenergic Agonists

Dexmedetomidine was evaluated in two preclinical studies for its potential to alleviate VIPN. The initial study demonstrated that dexmedetomidine, both alone and in combination with ulinastatin, significantly mitigated neuropathic pain induced by vincristine. The ED_50_ for dexmedetomidine in mitigating VIPN was 3.60 µg/kg [64]. Another study by Park et al. showed that dexmedetomidine administered in doses of 12.5, 25, 50, and 100 µg/kg produced a dose-dependent reduction in mechanical and cold allodynia in a rat model of VIPN [73]. Due to its ability to modulate pain pathways via α2-adrenergic receptor activation, dexmedetomidine is widely used in clinical anesthesia for its sedative and analgesic properties [74]. Growing evidence suggests that dexmedetomidine provides neuroprotective benefits through various mechanisms, including its antioxidative and anti-inflammatory properties, inhibition of apoptosis, promotion of neurogenesis, and regulation of cell signaling pathways [75].

#### 3.1.7. Histamine Receptor Antagonists and Mast Cell Stabilizer

Mast cells, originating from hematopoietic progenitor cells, mature after migrating to various tissues [76]. These inflammatory cells are found throughout the body, including brain regions such as the pituitary stalk, pineal gland, and hypothalamus [77]. They degranulate in response to immunological and non-immunological stimuli, releasing inflammatory mediators like histamine and cytokines [78]. These mediators also activate nociceptors on nerve endings, exciting nerve fibers [79]. Studies suggest that mast cells play a significant role in various neuropathic pain models, including those induced chemically [80,81,82]. Sodium cromoglycate inhibits mast cell degranulation and the release of inflammatory mediators, such as biogenic amines. Given that histamine is the main biogenic amine released during mast cell degranulation [77], researchers have investigated the role of mast cell-derived histamine in various pathological conditions using H1 and H2 receptor antagonists [83,84]. Building on this, Jaggi et al. [85] investigated the role of mast cells and mast cell-derived histamine in VIPN. Vincristine was administered over 10 days with a 2-day break to induce neuropathic pain. Over a 12-day period, sodium cromoglycate, the H1 antagonist promethazine, and the H2 antagonist ranitidine were administered, all demonstrating significant, dose-dependent pain reduction. Ranitidine was less effective, indicating a greater involvement of H1 receptors compared to H2 receptors in VIPN. These findings suggest that vincristine induces mast cell degranulation, releasing histamine that triggers pain through H1 and H2 receptors. 

#### 3.1.8. Antiemetics

The 5-HT3 receptor antagonist tropisetron has been explored for its potential to reduce VIPN through its effects on the serotonergic system. Barzegar-Fallah et al. administered tropisetron in a dose of 3 mg/kg i.p. one hour before vincristine treatment and observed a significant reduction in pro-inflammatory cytokines (TNF-α and IL-2 (interleukin 2)) and prevention of sensory-motor neuropathy [86]. Recent studies have also revealed its notable anti-inflammatory, immunomodulatory, and antioxidative properties, underscoring its therapeutic potential beyond emesis control.

#### 3.1.9. Antipsychotics

Aripiprazole is a novel quinolinone derivative with high lipid solubility, primarily employed clinically to treat mental disorders such as schizophrenia and bipolar disorder [87]. Acting as a partial agonist at 5-HT1A and dopamine D2 receptors, aripiprazole has shown a dose-dependent ability to mitigate PGE2 (prostaglandin E2)-induced hyperalgesia in the mechanical paw withdrawal test [88]. Aripiprazole was tested in a rat model of VIPN. Rats treated with aripiprazole (3 mg/kg i.p.) showed significantly higher mechanical withdrawal thresholds and improved sensory nerve conduction velocity (SNCV) compared to untreated controls. Additionally, aripiprazole reduced neuronal nitric oxide synthase (nNOS) expression and NF-kB (nuclear factor kappa B) activation in the dorsal root ganglia [89].

#### 3.1.10. Wakefulness-Promoting Agents

The psychostimulant modafinil was investigated for its ability to prevent VIPN. Pretreatment with modafinil (25 and 50 mg/kg) significantly increased response latencies in the tail-flick test (25 and 50 mg/kg), improved sensitivity in the von Frey test (50 mg/kg), and enhanced motor nerve conduction velocity (MNCV) (25 mg/kg). It also reduced levels of TRPA1 (transient receptor potential ankyrin 1), TNF-α, and IL-1β (interleukin 1 β), suggesting that modafinil’s analgesic effects are mediated by the suppression of inflammatory mediators and ion channel modulation [90]. Modafinil and its derivatives have demonstrated antioxidative, anti-inflammatory, and neuroprotective properties [90]. It enhances the activity of noradrenergic, dopaminergic, serotonergic, glutamatergic, and hypocretin (orexin) neurotransmitter systems while reducing GABA activity across diverse brain regions [91].

#### 3.1.11. Analgesics

Nefopam has garnered interest for its analgesic and antihyperalgesic effects, acting on monoamines and the noradrenergic and/or serotonergic systems to prevent and reduce central neural sensitization [92,93]. In a study using spinal nerve ligation models, intrathecal nefopam exhibited an antiallodynic effect by inhibiting the activation of microglia and astrocytes, which are known to release nociceptive mediators and facilitate the pain pathway [94]. Mice with VIPN allodynia were administered various doses of nefopam (10, 30, 60 mg/kg). The treatment resulted in a dose-dependent reduction in allodynia and a decrease in neurokinin 1 (NK1) receptors in the dorsal root ganglia. These results suggest that nefopam induces antinociception by lowering NK1 receptor levels, which in turn inhibits substance P and NK1 receptor signaling [95].

#### 3.1.12. IL-1 (Interleukin-1) Receptor Antagonists

Anakinra, a recombinant IL-1 receptor antagonist used clinically for rheumatoid arthritis [96], was tested for its potential to alleviate VIPN. Starobova et al. [97] demonstrated that vincristine activates IL-1β release from macrophages via the NLRP3 (NOD-like receptor family pyrin domain-containing 3) signaling pathway. Moreover, VIPN did not develop in mice deficient in IL-1 receptors or IL-1β. Anakinra administration (100 mg/kg) prevented the onset of vincristine-induced mechanical allodynia without impairing vincristine’s anticancer efficacy.

#### 3.1.13. Antibiotics

Minocycline, a tetracycline antibiotic, is traditionally used for the acute treatment of bacterial infections and for the long-term management of acne. Beyond its antimicrobial effects, minocycline exhibits several additional mechanisms of action that may be beneficial for treating non-infectious conditions [98]. While the precise anti-inflammatory and anti-apoptotic mechanisms of minocycline remain unclear, it has been observed to affect the activation of microglia and immune cells, along with the release of cytokines, chemokines, and nitric oxide [99,100]. Starobova et al. [101] aimed to identify new therapeutic targets for VIPN using a combination of behavioral, histological, and pharmacological approaches. Their findings revealed that local intraplantar injection of vincristine led to dose- and time-dependent mechanical hypersensitivity, which progressed to mechanical hyposensitivity at higher doses, accompanied by significant immune cell infiltration at the injection site. Notably, minocycline (25 mg/kg) effectively prevented both mechanical hypersensitivity and immune cell infiltration in two distinct mouse models of VIPN. The findings imply that minocycline’s effects may result from the inhibition of Toll-like receptor 4 (TLR4), as treated TLR4-deficient (Tlr4^−/−^) animals showed reduced hind paw sensitivity, decreased paw swelling, and less leukocytic perivascular infiltration.

#### 3.1.14. Selective Estrogen Receptor Modulators (SERMs)

Tamoxifen is a SERM and an inhibitor of protein kinase C (PKC), commonly prescribed for treating estrogen-positive breast cancer [102]. Tamoxifen was evaluated for its potential to alleviate VIPN in two preclinical studies. In the first study, oral administration of tamoxifen (1 mg/kg) every two days, starting one hour before vincristine injections and continuing for a total of seven doses, led to increased withdrawal latencies compared to vincristine alone. Additionally, tamoxifen treatment resulted in reduced spinal nitric oxide (NO) and serum TNFα levels [103]. Additionally, Tsubaki et al. [104] discovered that tamoxifen (30 mg/kg) effectively alleviates cold and mechanical allodynia induced not only by vincristine but also by paclitaxel and bortezomib in mice. The study emphasized that chemotherapy drugs trigger neuropathy via the PKC (protein kinase C)/ERK (extracellular signal-regulated kinase) pathway in the spinal cord, especially within lumbar segments 4–6 and in the dorsal root ganglia. Consequently, tamoxifen was found to alleviate chemotherapy-induced neuropathy by inhibiting the PKC/ERK pathway activation in these regions.

PKC activation results in elevated expression of phosphorylated ERK1/2 in the spinal cord, a process associated with chronic pain [105,106]. The sensitization of TRPV1 (transient receptor potential vanilloid 1) and TRPA1 (transient receptor potential ankyrin) channels, driven by PKCε activation, has been linked to paclitaxel-induced neuropathy, manifesting as cold and mechanical allodynia [107]. Furthermore, phosphorylation of PKC isoforms α, δ, and ε in the spinal cord and dorsal root ganglion has been observed in VIPN. These findings suggest that neuropathies caused by paclitaxel, vincristine, and bortezomib may be linked to the activation of transient receptor potential (TRP) channels mediated through PKC signaling [104].

#### 3.1.15. Antimigraine

Sumatriptan, a serotonin 5-HT1B/1D receptor agonist, was the first drug in its class developed for the acute treatment of migraines and continues to be widely used for this indication [108]. Sumatriptan reduces the release of neurotransmitters and neuropeptides, such as calcitonin gene-related peptide (CGRP) and substance P, by acting on serotonin receptors at the terminal end of neurons. Both neuropeptides counteract inflammatory factors effectively [109]. Khalilzadeh et al. [110] investigated the effect of sumatriptan in an animal model of VIPN. In the treatment group, sumatriptan (1 mg/kg) was administered i.p. 30 min before each VCR injection. Co-administration of sumatriptan with VCR significantly improved the hot plate, tail flick threshold, and sciatic MNCV tests, indicating prevention of mixed sensory-motor neuropathy. Sumatriptan also mitigated VCR-induced weight loss. Moreover, the treatment group showed significantly reduced levels of TNF-α, IL-1β, NF-κB, and caspase-3.

#### 3.1.16. Vasoprotective Agents

Theoesberiven F, a combination of proxyphylline and Melilotus extract, provides anti-inflammatory, anti-edematous, and analgesic effects. It is clinically prescribed for inflammatory conditions, edema, and circulatory disorders [111]. In addition, melilotus extract has anti-inflammatory and antioxidant properties, reducing thermal injury in rats by acting on phagocytic cells at the injury site [112]. The anti-allodynic effect of Theoesberiven F on mechanical and cold allodynia was studied in a rat model of VIPN. Male Sprague-Dawley rats received i.p. vincristine injections for 12 days. Allodynia was measured before and at intervals up to 180 min post-administration. The results showed that Theoesberiven F (0.1, 0.25, and 0.5 mg/kg) increased the paw withdrawal threshold in a dose-dependent manner and reduced the withdrawal frequency to cold stimuli induced by vincristine at doses of 0.25 and 0.5 mg/kg [111]. 

#### 3.1.17. Gastrin Receptor Antagonists

Netazepide, the gold standard for gastrin/CCK2R (cholecystokinin 2 receptor antagonists), has been tested in multiple trials for treating gastric neuroendocrine tumors and other conditions related to hypergastrinemia [113]. CCK2R is part of the CCKergic (cholecystokininergic) system, and it is primarily located in gastric enterochromaffin-like cells and in brain regions involved in pain modulation, memory, anxiety, and thermoregulation [114,115,116]. Research on CCK2R expression in the peripheral nervous system has revealed that CCK2R RNA is normally present in the dorsal root ganglion and becomes overexpressed after traumatic nerve injuries [115,117]. Preclinical studies have demonstrated the potential of CCK2R blockade for managing pain [116,118]. Bernard et al. [119] explored the effects of preventive CCK2R blockade with netazepide in a mouse model of VIPN. Mice received vincristine for 7 days to induce neuropathy, with netazepide (2 or 5 mg/kg/day) given starting one day before vincristine treatment. Vincristine caused significant tactile allodynia, dorsal root ganglion neuron and intraepidermal nerve fiber loss, and damage to myelinated axons. Netazepide effectively prevented these painful symptoms and nerve damage in a dose-dependent manner.

#### 3.1.18. Hormones

Oxytocin, a hormone critical for lactation and childbirth [120], has been investigated for its potential therapeutic effects in VIPN. Oxytocin treatment in rats reduced axonal damage, lipid peroxidation, and preserved NGF levels, highlighting its neuroprotective role [53]. Oxytocin also provides cytoprotection through its antioxidant, antiapoptotic, and anti-inflammatory effects [121,122].

Melatonin, a neurohormone with antioxidant and anti-inflammatory properties, primarily works by inhibiting specific calcium channels, targeting the α2δ1 protein subunit [123]. It can alleviate CIPN by reducing hypersensitivity and preserving nerve function [124]. In VIPN, melatonin reduced thermal hypersensitivity in the tail immersion test. Furthermore, melatonin shows significant biochemical effects at various dosages: at 5 mg/kg, it increases serum creatinine and urea levels, while at 10 mg/kg, it enhances catalase (CAT) activity and decreases urea levels. Additionally, melatonin reduces ALT, AST (aspartate aminotransferase), and MDA levels, suggesting a protective role against oxidative stress and liver damage. The combination of vincristine and melatonin may offer a strong synergistic effect, enhancing anticancer cytotoxicity and protecting the sciatic nerve from degeneration. Furthermore, melatonin has demonstrated synergistic protective and analgesic effects when used with pregabalin in vincristine-induced hypersensitivity [39].

The table below (Table 1) summarizes the results from preclinical studies discussed in this section, which evaluated pharmaceutical drugs and their mechanisms involved in alleviating VIPN. The studies are arranged in chronological order.

### 3.2. Natural Extracts and Compounds

Numerous natural extracts and compounds were evaluated for their potential to treat VIPN in animal models, aiming to identify alternative therapies that can effectively alleviate neuropathic symptoms while minimizing the side effects often associated with conventional treatments. These agents were categorized into three primary groups based on their predominant mechanisms of action: antinociceptive compounds, antioxidant compounds, and multi-target compounds.

#### 3.2.1. Antinociceptive Compounds

These compounds include various extracts traditionally used in herbal medicine, though their specific mechanisms of action in the context of VIPN are not fully defined. *Salvia officinalis* leaf extract, for example, effectively mitigated the pain response caused by vincristine, which notably increased the second phase of pain in VIPN mice [125]. Similarly, *Matricaria chamomilla*, a versatile herb valued for its sedative, pain-relieving, antispasmodic, anticonvulsant, anti-inflammatory, and wound-healing properties, has shown significant efficacy in reducing VIPN in mice models [126]. *Ginkgo biloba*, widely known for its protective effects on the nervous and circulatory systems, produced a dose-dependent antihyperalgesic effect in a rat model of VIPN, reducing sensitivity to mechanical and cold stimuli [127]. Another natural compound that showed promising results in reducing VIPN is the fruit of *Xylopia aethiopica*, commonly known as African pepper. A study by Woode et al. [128] demonstrated that the ethanolic extract of the fruit, along with its major diterpene component, has potent anti-hyperalgesic, tactile, and cold anti-allodynic effects. *Palisota hirsuta* extract, recognized for its antinociceptive and antidepressant properties, administered in different doses (30–300 mg/kg) significantly reduced pain-related behaviors at doses of 100–300 mg/kg in a VIPN model [129]. Likewise, *Synedrella nodiflora* extract significantly reversed tactile allodynia at doses of 100 and 300 mg/kg, cold allodynia at doses of 300 mg/kg, and mechanical hyperalgesia in a dose-dependent manner, underscoring its potential neuroprotective properties [130]. Recent studies have also highlighted the efficacy of *Tithonia tubaeformis* extract, which effectively alleviated vincristine-induced allodynia and thermal hyperalgesia in rats at doses of 100 mg/kg and 200 mg/kg, suggesting its promise as a natural therapeutic option for VIPN [131].

#### 3.2.2. Antioxidant Compounds

These compounds primarily act by reducing oxidative stress markers and enhancing endogenous antioxidant defenses, thereby preserving neuronal function and integrity. *Acorus calamus* rhizome extract, known for its antioxidant, anti-inflammatory, and neuroprotective properties, successfully prevented VIPN in rats in a dose-dependent manner. [132]. Butea monosperma, traditionally used to treat inflammation and other conditions, administered in different doses (200, 300, and 400 mg/kg) reversed vincristine-induced behavioral changes and corrected biochemical imbalances, such as elevated TBARS (thiobarbituric acid reactive substances) and calcium levels, while restoring GSH (glutathione) levels in a dose-dependent manner [133]. Similarly, *Vernonia cinerea*, a plant widely used in traditional medicine for treating infections, wound healing, and gastrointestinal issues, administered in different doses (200, 300, and 400 mg/kg) significantly alleviated VIPN in a dose-dependently and normalized biochemical alteration through its antioxidative, neuroprotective, and calcium channel-modulating properties [134]. *Ocimum sanctum*, an Indigenous herb highly regarded in Ayurveda for its diverse therapeutic benefits and its saponin-rich fraction, administered over 14 days, also demonstrated the ability to alleviate neuropathic pain, reduce oxidative stress, and lower calcium levels [135]. Finally, curcumin, a well-known antioxidant, significantly improved antioxidant levels and total calcium levels in vincristine-treated mice, effectively alleviating a variety of pain symptoms associated with VIPN at doses of 60 mg/kg [136].

#### 3.2.3. Multi-Target Compounds

These agents exert their therapeutic effects through a combination of mechanisms, including antioxidant, anti-inflammatory, and neuroprotective mechanisms, providing comprehensive relief from VIPN symptoms.

*Tribulus terrestris* saponins demonstrated strong anti-inflammatory properties by significantly reducing levels of inflammatory mediators in the sciatic nerve and brain, along with decreasing excitatory neurotransmitters such as L-glutamic acid and L-aspartic acid, suggesting a restoration of neuronal function and synaptic activity [137]. Gastrodin, a natural compound traditionally used as an analgesic [138], alleviated pain in VIPN rats by modulating sodium channel functions and reducing the expression of pro-inflammatory factors like TNF-α and IL-1β. Additionally, it inhibited spinal microglial activation via the CX3CL1 (fractalkine)/CX3CR1 (C-X3-C motif chemokine receptor 1) axis, subsequently suppressing the P38/MAPK (mitogen-activated protein kinase) signaling pathway [139,140,141]. CX3CL1 and its receptor CX3CR1 are essential in activating spinal microglia, with Iba-1 (ionized calcium-binding adapter molecule 1) serving as a marker for microglial activation [142]. High expression of CX3CL1 and CX3CR1 triggers microglia to release inflammatory factors like TNF-α and IL-1β via the P38/MAPK signaling pathway, contributing to CIPN [143].

Matrine (15, 30, and 60 mg/kg) also showed an antinociceptive effect dose-dependently, reducing oxidative stress and exerting anti-inflammatory actions [144,145]. Additionally, tetrahydrocurcumin, a derivative of curcumin, attenuated VIPN symptoms at doses of 80 mg/kg through its multi-faceted actions, which include anti-nociceptive, anti-inflammatory, neuroprotective, calcium-inhibitory, and antioxidant properties [146]. Repeated doses of fucoidan (50, 100, or 200 mg/kg), a complex sulfated polysaccharide derived from marine brown seaweed and recognized for its anticoagulant, antithrombotic, antiviral, antitumor, antioxidant, and anti-inflammatory properties [147,148], reduced vincristine-induced mechanical and cold allodynia in a dose-dependent manner by upregulating GABA-B receptor expression [149]. Morin (25, 50, and 100 mg/kg) mitigated sciatic nerve deficits and reduced neuronal hyperexcitability by decreasing key inflammatory markers such as IL-6 (interleukin 6) and NF-κB, thereby providing both anti-inflammatory and neuroprotective benefits in a dose-dependent manner [150].

In a recent study, bergapten, a furocoumarin from the *Rutaceae* family, regulated NF-κB activity, lowered plasma levels of TNF-α and IL-1β, and reduced the expression of iNOS (inducible nitric oxide synthase) and COX-2 (cyclooxygenase-2) in the spinal cord and sciatic nerve, thereby reversing vincristine-induced neuropathic pain [151]. 5,7-dimethoxycoumarin reversed vincristine-induced thermal hyperalgesia in a dose-dependent manner, mechanical allodynia, and cold allodynia at doses of 40 and 50 mg/kg, restoring neurotransmitter levels such as serotonin and dopamine [152].

Levo-corydalmine targeted multiple pathways, including the Nrf2 (nuclear factor erythroid 2–related factor 2)/HO-1 (heme oxygenase-1)/CO (carbon monoxide) axis, reducing inflammation and pain while modulating oxidative stress and apoptosis-related proteins [153]. The expression of HO-1 is controlled by multiple transcription factors, including NF-κB and Nrf2 [154]. In cases of VIPN, Nrf2 directly binds to sequences upstream of the HO-1 promoter, facilitating heme degradation and CO production. Nrf2 activation inhibits NF-κB activity, and conversely, NF-κB can suppress Nrf2, highlighting a cross-talk mechanism where NF-κB activation and Nrf2 inhibition contribute to neuroinflammation [155,156]. Levo-corydalmine mitigates this effect by inhibiting NF-κB while simultaneously increasing Nrf2 expression, effectively reducing the neuroinflammatory response induced by vincristine. Activation of the HO-1/CO pathway has been demonstrated to relieve both acute and chronic inflammatory pain, including pain associated with diabetic neuropathy and neuropathy induced by chronic constriction injury [157,158]. The results of the study further demonstrate that Levo-corydalmine increases CO release in the spinal cord and cultured astrocytes in a dose-dependent manner [153].

In a study investigating its potential impact on VIPN, puerarin, the main active component of *Puerariae lobatae* radix, was administered orally at two dose levels (25 or 50 mg/kg/day) for three weeks. The results revealed that puerarin effectively alleviated hyperalgesia and allodynia and restored levels of TNF-α and IL-1β while increasing levels of TGF-β (transforming growth factor-beta) and IL-10. On a molecular level, puerarin treatment downregulated the protein expression of IL-1β and NF-κBp65 while upregulating TGF-β, p-Smad2 (phosphorylated mothers against decapentaplegic homolog 2), and p-Smad3 (phosphorylated (mothers against decapentaplegic homolog 3) in the spinal cord and dorsal root ganglia [159]. Smad (Sma and Mad-related protein) transcription factors are the primary effectors of the TGF-β family, mediating a range of effects both in vitro and in vivo [160]. Activating Smad signaling in the dorsal root ganglion has been found to promote sensory axon regeneration in spinal cord injury models [161]. The previous study observed that expressions of p-Smad2, p-Smad3, and TGF-β proteins were downregulated following vincristine injection [159].

Finally, withametelin, derived from *Datura innoxa*, showed potent antinociceptive effects by modulating various signaling pathways, such as TRPV1 (transient receptor potential vanilloid 1)/TRPM8 (transient receptor potential melastatin 8)/P2Y, MAPK, and Bax (Bcl-2-associated X protein)/Bcl-2 (B-cell lymphoma 2)/caspase-3 in the spinal cord [162]. TRP channels, particularly TRPV1 and TRPM8, are critical players in chemotherapy-induced mechanical and thermal hypersensitivity [163]. TRPV1 is mainly linked to hypersensitivity to thermal, chemical, and mechanical stimuli related to peripheral nerve damage. In contrast, increased TRPM8 expression in the spinal cord and dorsal root ganglion has been observed in animal models of neuropathic pain, highlighting TRPM8 as a potential target for pain management [164]. The P2Y receptor is also implicated in neuropathic pain, with its inhibition shown to relieve pain hypersensitivity in animal models [165]. MAPK signaling, which includes ERK and p38-MAPK, is activated in the spinal dorsal horn following peripheral nerve injury and plays a key role in neuropathic pain by regulating the transcription of both P2Y and TRPV1 receptors [166,167,168,169]. JNK (c-Jun N-terminal kinase) signaling is also essential in apoptosis, where it deactivates anti-apoptotic Bcl-2 proteins and activates pro-apoptotic proteins like Bax and caspase-3, thereby accelerating cell death in response to vincristine-induced peripheral nerve damage [170]. The upregulation of Bax/caspase-3 and downregulation of Bcl-2 confirm the activation of apoptotic pathways, exacerbating nerve damage and pain [171].

Thymoquinone (2.5, 5, and 10 mg/kg), a bioactive compound from *Nigella sativa*, improved pain responses in a dose-dependent manner and reduced oxidative stress and inflammatory markers, indicating its potential as a versatile therapeutic agent in VIPN [172]. Finally, L-theanine, a compound found in tea leaves, significantly improved pain thresholds, enhanced antioxidant defenses, and reduced neuroinflammatory activity, making it a promising candidate for managing VIPN through its multi-targeted effects [173].

Table 2 summarizes in chronological order the results from preclinical studies discussed in this section, which evaluated natural extracts and compounds and their mechanisms involved in alleviating VIPN.

### 3.3. Antioxidants and Vitamins

Thioctic acid, a sulfur-containing antioxidant derived from caprylic acid, is essential for aerobic metabolism [174], was studied in a rat model of neuropathy induced by vincristine injections. After 15 days, rats with mechanical and cold allodynia were treated with either normal saline or thioctic acid (1, 5, or 10 mg/kg). Allodynia was evaluated before and at intervals (15, 30, 60, 90, 150, and 180 min) after treatment. Results demonstrated that thioctic acid effectively reduced both mechanical and cold allodynia, with the effect becoming more pronounced at higher doses [175].

Ferulic acid, a phytochemical known for its potent antioxidant properties, commonly found in fruits and vegetables like corn bran, wheat bran, eggplant, and citrus fruits [176], was studied by Vashistha et al. [177] for its effects on VIPN in rats. Pain sensitivity was assessed using pinprick, hot plate, paint-brush, and acetone tests on days 1, 6, 14, and 21, while inflammation and oxidative stress were measured through markers like TBARS, GSH, MPO, TNF-alpha, IL-6, IL-10, and total calcium. Ferulic acid, administered at doses of 50 and 100 mg/kg over 11 days, significantly reduced vincristine-induced behavioral changes, oxidative stress, and inflammation, demonstrating its potential therapeutic benefits in managing VIPN. Studies showed that ferulic acid can protect rats from oxidative stress-related apoptosis following cerebral ischemia/reperfusion injury [178]. Additionally, another research suggested that it may alleviate neuropathic pain caused by chronic constriction injury by targeting the P2X3 receptor [179].

Coenzyme Q10, acting as a vital endogenous antioxidant [180], was shown to reduce thermal hypersensitivity in both tail immersion and acetone tests, significantly improve oxidative stress and inflammatory biomarkers, decrease serum NFL (neurofilament light chain) levels, enhance Nrf2 expression, and downregulate SARM1 (sterile alpha and TIR motif containing 1) gene expression [181]. SARM1 plays a central role in axonal degeneration by activating the MAPK pathway and depleting NAD+ levels [182]. Elevated ROS levels can trigger SARM1, leading to axon degradation and cell death in a process known as “sarmoptosis,” a unique cell death mechanism distinct from apoptosis. This discovery adds a significant new perspective to the molecular understanding of programmed cell death [183]. Mitoquinone, a derivative of Coenzyme Q, significantly alleviated vincristine-induced pain hypersensitivity, glial activation, and oxidative stress in the spinal cord by enhancing Nrf2 expression. Mitoquinone also reduced pro-inflammatory cytokine levels in a dose-dependent manner, highlighting its anti-inflammatory properties. Furthermore, mitoquinone effectively mitigated neuron death and mitochondrial dysfunction caused by vincristine, as evidenced by reduced cleaved caspase-3 and Bax levels, and restored Bcl-2. Additionally, mitoquinone inhibited cytochrome C release from mitochondria.

Vitamin E is a key antioxidant that protects neuronal tissue from oxidative damage and is the primary lipid-soluble chain-breaking antioxidant in the body [184]. The neuroprotective effects of vitamin E were assessed in albino rats. After administering vitamin E, rats received vincristine on alternate days for 14 days to induce peripheral neuropathy. Vitamin E at doses of 100 mg/kg and 200 mg/kg significantly reduced reaction times on days 7 and 14 across all three tests compared to the control group [184].

Methylcobalamin, a cobalt-containing form of vitamin B12 with a high affinity for nerve tissues [185], dose-dependently alleviated vincristine-induced allodynia and hyperalgesia, enhanced intraepidermal nerve fiber density, and reduced the prevalence of atypical mitochondria in the sciatic nerve. Methylcobalamin also inhibited NADPH oxidase activation and the NF-κB pathway. Additionally, it decreased TNF-α production and increased IL-10 levels in the spinal dorsal horn [186].

Table 3 provides a summary of the preclinical study results discussed in this section, focusing on the evaluation of antioxidants and vitamins, along with their mechanisms in alleviating VIPN.

## 4. Discussion

Our narrative review focused on summarizing the potential effects of different chemical and natural substances in animal models of VIPN. Vincristine is a widely used chemotherapy drug, but its effectiveness is often limited by its dose-dependent neurotoxicity. This can necessitate treatment discontinuation and negatively impact patient health [188,189,190,191]. CIPN, particularly VIPN, involves a multitude of complex mechanisms that affect both neuronal function and structure.

### 4.1. Mechanistic Insights into VIPN

VIPN arises from multiple interrelated mechanisms. By attaching to β-tubulin, vincristine stops the movement of axons, which damages microtubules and causes sensory neurons to die [192,193,194,195]. Increased neuronal activity and genetic changes, such as the upregulation of ATF3 (activating transcription factor 3), heighten sensory sensitivity in VIPN [196]. Oxidative stress and IL-1 release trigger astrocyte activation, which amplifies NMDA receptor activity and increases pain perception [197,198,199]. Dysregulated sodium and calcium channels, along with potassium channel dysfunction, further raise neuron excitability [200,201,202,203]. Pro-inflammatory cytokines (TNF-α, IL-6, IL-1β) and chemokines (CXCL1 (chemokine ligand 1), CXCL12 (chemokine ligand 12)) drive neuroinflammation in VIPN. In addition, macrophage activation and Schwann cell damage worsen nerve injury and pain [64,197,204,205,206]. These multifactorial processes underscore the complexity of VIPN pathogenesis, making it challenging to develop targeted treatments.

### 4.2. Risk Factors for VIPN

**Figure 1:**
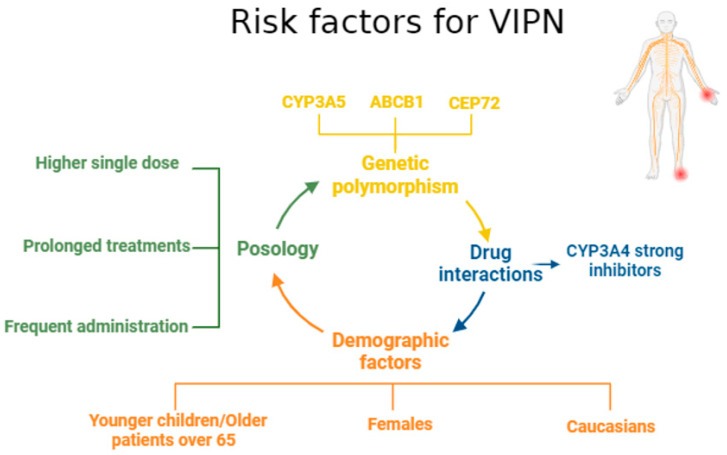
Risk factors for VIPN (ABCB1—ATP-binding cassette sub-family B member 1; CEP72—Centrosomal Protein 72, VIPN—vincristine-induced peripheral neuropathy; created with BioRender version, Bio Rad 2023).

Various risk factors influence the likelihood and severity of VIPN, including genetic, pharmacological, and demographic variables (Figure 1) [207,208,209,210,211,212,213,214,215].

Vincristine is metabolized mainly by the CYP3A4 and CYP3A5 enzymes, with CYP3A5 being more efficient [207,216,217,218]. Genetic variations in these enzymes affect vincristine metabolism and influence VIPN risk. For instance, the CYP3A53 variant, which is common in Caucasians, is linked to slower vincristine clearance and increased neurotoxicity. In contrast, African-Americans, who more frequently express active CYP3A5, show lower rates of neurotoxicity [219]. Additionally, genetic polymorphisms in ABCB1 (P-glycoprotein) and CEP72 (centrosomal protein of 72 kDa) also contribute to VIPN susceptibility [209,210,220,221,222,223,224]. Higher vincristine doses and prolonged treatments increase VIPN risk, especially in adults. Guidelines recommend capping single doses at 2 mg to minimize neurotoxicity [211,212]. Administering vincristine via prolonged infusions rather than bolus injections may reduce VIPN by optimizing drug clearance [225,226]. CYP3A4 inhibitors, like itraconazole, can raise the level of vincristine, which increases the risk of neurotoxicity [227,228,229]. Fluconazole, a weaker inhibitor, is preferred in patients requiring antifungal prophylaxis [230]. Aprepitant and fosaprepitant, used as antiemetics, also inhibit CYP3A4 and may exacerbate VIPN, requiring careful consideration during vincristine therapy [213,231]. Demographic factors also play a role. Younger children may be more vulnerable to VIPN due to immature nervous system development. Older patients, particularly those over 65, face a higher risk of neuropathy [232,233,234,235]. Female patients and Caucasians are also at greater risk of VIPN. Caucasians are more likely to experience neurotoxicity because their CYP3A5 expression is lower, but they have higher overall survival rates than African Americans [210,214,215,236,237].

### 4.3. Pharmacological Interventions for VIPN

Preclinical studies have explored a diverse range of pharmaceutical agents to alleviate VIPN. In the pharmaceutical domain, antiepileptics such as ethosuximide, lacosamide, topiramate, and zonisamide offered promising relief from pain [25,28,31]. Antidepressants like duloxetine effectively decreased pain sensitivity, aligning with its first-line status in CIPN management [10,42]. Similarly, NMDAR antagonists, such as amantadine, provided neuroprotection through anti-inflammatory and antioxidant pathways [3,46]. Metabolic agents such as simvastatin and liraglutide enhanced neuronal health and alleviated neuropathic pain [49,53]. Enzyme inhibitors like ulinastatin and cilostazol, along with propentofylline, provided anti-inflammatory benefits [59,61,64].

Alpha-2 adrenergic agonists (dexmedetomidine), mast cell stabilizers (sodium cromoglycate), and antiemetics (tropisetron) further demonstrated efficacy in reducing VIPN symptoms through unique mechanisms [73,85,86]. The psychostimulant modafinil, analgesic nefopam, and IL-1 receptor antagonist anakinra also showed potential, while agents like aripiprazole and tamoxifen acted on inflammatory and receptor pathways to alleviate VIPN [89,90,95,97,103].

### 4.4. Natural Remedies in VIPN

Natural compounds showed significant promise in alleviating VIPN through multifaceted mechanisms. Ginkgo biloba and Matricaria chamomilla provided notable pain relief [126,132]. Acorus calamus and Butea monosperma offered dose-dependent pain relief, also providing antioxidant benefits [127,133]. Gastrodin, widely used as an analgesic in traditional medicine, mitigated hyperalgesia through sodium channel regulation and anti-inflammatory pathways [134]. Vernonia cinerea alleviated hyperalgesia by leveraging its antioxidative, neuroprotective, and calcium channel-modulating properties [139]. Xylopia aethiopica and matrine had strong pain-relieving effects, and Ocimum sanctum and curcumin showed neuroprotective effects through their antioxidant activities [128,135,136,145]. Fucoidan, a sulfated polysaccharide, alleviated VIPN by enhancing GABA-B receptor expression and reducing inflammation [149]. Other notable agents include Tithonia tubaeformis, Tribulus terrestris, morin, bergapten, Levo-corydalmine, puerarin, withametelin, thymoquinone, and L-theanine, each providing comprehensive symptom relief through unique multi-targeted actions [131,137,150,151,153,159,162,172,173]. 

### 4.5. Role of Antioxidants and Vitamins in VIPN

Antioxidants and vitamins play a crucial role in alleviating VIPN by targeting oxidative stress and inflammation. Compounds such as ferulic acid, coenzyme Q10, and mitoquinone have shown significant potential in reducing pain sensitivity in preclinical studies. Additionally, coenzyme Q10 and mitoquinone have been found to lower oxidative stress, modulate inflammatory pathways, and support neuronal health [108,110,114].

Among vitamins, methylcobalamin has shown neuroprotective effects by reducing TNF-α levels, increasing IL-10 levels, and preserving nerve fiber density, making it a strong candidate for VIPN management [113]. Vitamin E effectively attenuated pain sensitivity, highlighting its potential in alleviating VIPN symptoms [111].

### 4.6. Limitations and Future Perspectives

The mechanisms and effects of various substances on VIPN in animal models reveal that these compounds target multiple pathways to alleviate symptoms. Most agents act by modulating oxidative stress, ion channels (calcium, sodium, NMDA), neurotransmitter systems (GABA, serotonin, norepinephrine, glutamate), cytokine and inflammatory pathways (IL-1 and TNF-α), and specific receptors (histamine, 5-HT, and NK1). By targeting these diverse pathways, the compounds work to reduce neuronal excitability, inhibit pain signaling, and decrease inflammation, ultimately providing symptomatic relief from VIPN. This wide range of mechanisms highlights the complex nature of VIPN and underscores the need for a multifaceted therapeutic approach.

According to ASCO guidelines, duloxetine is the only clinically endorsed treatment for CIPN due to its proven efficacy in pain reduction. However, limitations such as patient tolerance and the potential for withdrawal symptoms upon abrupt discontinuation highlight a need for complementary treatments **[10]**. Agents like thioctic acid and curcumin, shown in animal models to reduce neuropathic pain and oxidative stress, could be evaluated in combination with duloxetine. By targeting different neuropathic pathways, these agents might enhance duloxetine’s effects or offer a substitute in cases where duloxetine is not well tolerated. Moreover, compounds such as *Ginkgo biloba* and *Acorus calamus* showed neuroprotective benefits in preclinical studies and could serve as adjunct therapies, potentially mitigating symptoms duloxetine may not fully address. ASCO guidelines now suggest caution with tricyclic antidepressants and gabapentinoids due to limited CIPN-specific efficacy and unfavorable side effects [10]. Other antiepileptics like lacosamide and zonisamide have shown beneficial effects in animal models and could make them viable alternatives, warranting further investigation to determine their role in managing VIPN. Inconsistent results and lack of FDA approval limit the use of baclofen, amitriptyline, and ketamine topical gels [10]. Natural compounds like *Vernonia cinerea* and *Tithonia tubaeformis*, both demonstrating anti-inflammatory and analgesic properties in animal studies, could be explored as alternative topical treatments. Given that natural extracts generally carry fewer regulatory hurdles, they might offer more accessible and less complex options for topical neuropathy management, especially if developed as standardized FDA-approved products. ASCO’s guidelines favor duloxetine over gabapentinoids; however, insurance policies often mandate the use of gabapentinoids. This policy leads to gabapentinoids being prescribed more frequently than duloxetine, despite ASCO’s recommendations [10]. Highlighting preclinical alternatives supports guideline updates that reflect the latest efficacy data. Integrating these findings into clinical trials could expand CIPN treatments and improve patient outcomes, especially for those who do not tolerate duloxetine.

Despite extensive preclinical research on agents with potential efficacy in alleviating VIPN, clinical data remain limited. Jeenia et al. demonstrated that vitamins B6 and B12 were effective in significantly reducing the incidence, relative risk, and severity of VIPN in a study with 81 patients [238]. In a recent study, Angelescu et al. evaluated the effects of gabapentin at a dosage of 20 mg/kg on 49 children aged 1 to 18 years. The findings indicated that both opioid consumption and pain scores were higher in the gabapentin group compared to the placebo group [239]. Glutamine supplementation was assessed in children and adolescents with VIPN, yielding promising outcomes. The findings showed that glutamine is well tolerated and linked to improvements in sensory function and self-reported overall quality of life [240]. Despite promising insights from preclinical studies, significant challenges remain in translating these findings into clinical practice. Animal models, though valuable, often fail to accurately replicate human physiology and disease pathology, leading to discrepancies in treatment efficacy and safety. Variations in dosages, administration routes, and experimental designs across studies further complicate efforts, making it difficult to establish standardized protocols. While natural compounds, such as *Ginkgo biloba*, *Acorus calamus*, and curcumin, have shown promising neuroprotective effects in rodent models of VIPN, the transition to human applications is complex. Species-specific differences in metabolism, absorption, and dosage scaling can significantly influence therapeutic efficacy and safety in clinical contexts. Furthermore, the complexity of human neuropathic pain mechanisms, shaped by genetic, environmental, and lifestyle factors, is not fully represented in animal models, potentially limiting the clinical relevance of these findings. To improve the translational potential of preclinical research, we recommend refining animal models to better reflect human pathophysiology and establishing standardized preclinical protocols for dosage and administration. This will enhance data consistency, facilitate cross-study comparisons, and ultimately accelerate the translation of promising therapies into clinical use. Moreover, combination therapies that target multiple pathways, such as neuroinflammation, oxidative stress, and ion channel regulation, warrant further exploration, with particular emphasis on calcium and sodium channel modulation, as well as key inflammatory cytokines (e.g., IL-1β, TNF-α). Future research should also incorporate pharmacokinetic and toxicity studies tailored to human physiology, along with early-phase clinical trials, to assess safety and dose tolerability. These rigorous evaluations are essential to determining whether the therapeutic benefits observed in animal studies can be reliably and safely reproduced in human patients, ultimately paving the way for more effective and viable treatment options. By addressing these challenges, we can help bridge the gap between preclinical promise and clinical applicability, advancing therapeutic options for conditions such as VIPN.

## 5. Conclusions

In conclusion, our review underscores the diverse and promising array of substances for alleviating pain associated with VIPN. Both pharmaceutical drugs and natural compounds have demonstrated encouraging outcomes through various mechanisms in preclinical studies. Despite the significant therapeutic potential of many new compounds, most are still in the preclinical stage. To fully assess their effectiveness and safety in managing VIPN, well-designed clinical trials are essential for the compounds reviewed.

## Figures and Tables

**Table 1:** Preclinical studies investigating the effects of pharmaceutical drugs on VIPN.

Tested Substances/Doses	Animals	Results	Involved Mechanism	Reference
Ethosuximide 300 mg/kg bw i.p.	Sprague Dawley rats	Reversed mechanical and thermal sensitivity in von Frey and acetone tests.	N/A	Flatters et al. (2004) [25]
Propentofylline 10 mg/kg bw i.p.	Holtzman rats	Reduced mechanical allodynia in von Frey test.	⇓ spinal microglial and astrocytic activation	Sweitzer et al. (2006) [61]
Lacosamide 3, 10, 20, 30, and 40 mg/kg bw i.p.	Sprague Dawley rats	Attenuated thermal allodynia in hot–cold plate tests and mechanical hyperalgesia in the Randall–Selitto test.	N/A	Beyreuther et al. (2007) [28]
Memantine 2.5, 5, and 10 mg/kg bw i.p.	Sprague-Dawley rats	The highest dose of memantine successfully increased the paw withdrawal threshold in the von Frey test.	N/A	Park et al. (2010) [46]
Dexmedetomidine 12.5, 25, 50, and 100 µg/kg bw i.p.	Sprague Dawley rats	Displayed a dose-dependent antiallodynic effect on mechanical and cold stimuli in von Frey, acetone, and hot plate tests.	N/A	Park et al. (2012) [73]
Tamoxifen 1 mg/kg bw orally	Egyptian rats	Attenuated thermal hypersensitivity in tail immersion and hot plate tests.	⇓ TNF-α ⇓ NO	El-Masry et al. (2012) [103]
Cilostazol 20 and 40 mg/kg bw orally	Albino Swiss mice	Attenuated mechanical hyperalgesia and mechanical allodynia in pinprick and paintbrush tests.	N/A	Thacheril Mohanan et al. (2013) [59]
Topiramate 40 mg/kg bw OR Zonisamide 50 mg/kg bw OR Gabapentin 60 mg/kg bw orally	Albino rats	All 3 drugs significantly reduced thermal hyperalgesia in the hot plate test.	N/A	Jakhar et al. (2014) [31]
Tropisetron 3 mg/kg bw i.p.	Sprague Dawley rats	Attenuated thermal sensitivity in the hot plate test.	⇑ MNCV ⇓ TNF-α ⇓ IL-2	Barzegar-Fallah et al. (2014) [86]
Duloxetine 10, 20, and 30 mg/kg bw orally	Albino rats	Reduced thermal hypersensitivity in hot plate and tail immersion tests. Increased ambulation, activity factor, and mobility duration in open field test.	⇑ NCV ⇓ CD11b	Greish et al. (2014) [43]
Duloxetine 5, 10, and 20 mg/kg bw *OR* Milnacipran 10, 20 and 40 mg/kg bw	ddY-strain mice	Both drugs significantly reduced mechanical allodynia in the von Frey test.	N/A	Katsuyama et al. (2014) [42]
Simvastatin 7.5, 15, and 30 mg/kg bw i.p.	Wistar rats	Attenuated thermal (acetone and hot plate tests) and mechanical (pinprick and von Frey tests) sensitivity only at lower doses.	⇓ MPO ⇓ cholesterol	Bhalla et al. (2015) [49]
Theoesberiven F 0.1, 0.25 and 0.5 mg/kg bw i.p.	Sprague Dawley rats	Reduced mechanical and cold allodynia in Von Frey and acetone tests.	N/A	Bang et al. (2016) [111]
Ulinastatin 0.5–5.0 × 10^5^ U/kg i.t. *OR* Dexmedetomidine 0.4–4.0 μg/kg i.p. *OR* Ulinastatin 0.81–1.75 × 10^5^ U/kg + Dexmedetomidine 0.69–1.47 U/kg μg/kg	Sprague Dawley rats	Both drugs attenuated mechanical sensitivity in the von Frey test. Dexmedetomidine and ulinastatin exhibited a synergistic effect when used together.	Alone and in combination: ⇑ IL-10	Nie et al. (2017) [64]
Sodium cromoglycate 10 and 20 mg/kg bw *OR* Promethazine 50 and 100 mg/kg bw *OR* Ranitidine 20 and 40 mg/kg bw i.p.	Wistar rats	All three drugs reduced pain sensitivity in pinprick, acetone, and hot plate tests, with a less pronounced effect for ranitidine.	N/A	Jaggi et al. (2018) [85]
Tamoxifen 30 mg/kg bw p.o.	Balb/c mice	Suppressed cold and mechanical allodynia in hot–cold plate test.	-inhibited PKC/ERK pathway	Tsubak et al. (2018) [104]
Sumatriptan 1 mg/kg bw i.p.	Sprague Dawley rats	Reversed thermal and mechanical sensitivity in hot plate, von Frey, and tail-flick tests.	⇑ MNCV ⇓ TNF-α ⇓ IL-1β ⇓ caspase-3 ⇓ NF-kB	Khalilzadeh et al. (2018) [110]
Minocycline 25 mg/kg bw i.p.	C57BL/6J AND Tlr4^−^/^−^ mice	Prevented mechanical allodynia in the von Frey test, and hind paw inflammation induced by vincristine in both models.	⇓ perivascular infiltrate ⇓ fibroblast reaction	Starobova et al. (2019) [101]
Aripiprazole 3 mg/kg bw i.p.	Wistar rats	Reduced pain sensitivity in hot plate and von Frey tests.	⇑ SNCV ⇓ nNOS ⇓ NF-kB	Khalilzadeh et al. (2020) [89]
Modafinil 5, 25, and 50 mg/kg bw i.p.	Wistar rats	Lowered the hyperalgesia in the tail-flick and von Frey tests.	⇑ MNCV ⇓ TNF-α ⇓ IL-1β ⇓ TRPA1	Amirkhanloo et al. (2020) [90]
Oxytocin 100 μg/kg bw *OR* Liraglutide 1 mg/kg bw i.p.	Sprague Dawley rats	Liraglutide was the only treatment that significantly restored muscle strength in the inclined-plate test.	Both drugs: ⇑ NGF ⇓ MDA	Erdogan et al. (2020) [53]
Nefopam 10, 30, and 60 mg/kg bw i.p.	Mice	Reversed mechanical allodynia in von Frey test.	⇓ neurokinin-1 receptor concentration	Lee et al. (2020) [95]
Melatonin 5 and 10 mg/kg bw Orally *OR* Pregabalin 10 mg/kg bw Orally *OR* Melatonin 5 mg/kg bw *AND * Pregabalin 15 mg/kg bw orally	Wistar rats	Reduced thermal hypersensitivity in tail immersion test when administered alone and in combination.	Melatonin: ⇑ serum creatinine (5 mg/kg) ⇑ CAT (10 mg/kg) ⇑ urea (5 mg/kg) ⇓ ALT ⇓ AST ⇓ urea (10 mg/kg) ⇓ MDA Pregabalin: ⇓ AST ⇓ MDA ⇑ urea Combination: ⇑ CAT ⇑ urea ⇓ AST ⇓ MDA	Soliman et al. (2020) [39]
Anakinra 100 mg/kg bw i.p.	C57BL/6J mice	Significantly attenuated mechanical hypersensitivity in von Frey test.	N/A	Starobova et al. (2021) [97]
Amantadine 2, 5, 12, 25, and 50 mg/kg bw orally	Wistar rats	Reduced pain sensitivity in digital analgesia meter test.	⇑ Bcl xl ⇑ CAT ⇑ SOD ⇑ IL-10 ⇓ IL-6 ⇓ TNF-α ⇓ MIP-1α ⇓ Perk gene expression ⇓ Bax ⇓ Casp 3 ⇓ Casp 9 ⇓ CX3CR1	Drummond et al. (2024) [3]
Netazepide 2 and 5 mg/kg bw orally	Swiss mice	Completely reversed mechanical hypersensitivity in the von Frey test.	-prevented the loss of DRG neurons, the reduction in myelinated fiber density, and the increase in myelinated axon	Bernard et al. (2024) [119]

⇑-increased; ⇓-decreased; ALT—alanine transaminase; AST—aspartate aminotransferase; Bax-Bcl2—associated X-protein; Bcl xl—B-cell lymphoma-extra large; bw—body weight; Casp 3—caspase 3; Casp 9—caspase 9; CAT—catalase; COX2—cyclooxygenase 2; CX3CR1—C-X3-C motif chemokine receptor 1; DRG—dorsal root ganglions; ERK—Extracellular Signal—Regulated Kinase; GSH-reduced glutathione; i.p.—intraperitoneally; IL-10—interleukin 10; IL-18—interleukin 18; IL-1β—interleukin 1β; IL-2—interleukin 2; LPO—lipid peroxidation; MDA—malondialdehyde ; MIP-1 α—macrophage inflammatory protein-1 alpha; MNCV—motor nerve conduction velocity; MPO—myeloperoxidase; N/A-not applicable; NADPH—nicotinamide adenine dinucleotide phosphate; NCV—nerve conduction velocities; NF-κB—nuclear factor kappa B; NGF—nerve growth factor; nNOS—neural nitric oxide synthase; NO—nitric oxide; Nrf2—NF-E2 related factor-2; PKC—protein kinase C; ROS—reactive species of oxygen; SNCV—sciatic nerve conduction velocity; SOD—superoxide dismutase; TNF-α—tumor necrosis factor α; TRPA1—transient receptor potential cation channel ankyrin 1; α2-AR—α2-adrenergic receptor.

**Table 2:** Preclinical studies investigating the effects of natural extracts and compounds on VIPN.

Tested Substances/Doses	Animals	Results	Involved Mechanism	Reference
*Salvia officinalis* extract 100 mg/kg bw i.p.	NMRI mice	Significantly decreased pain in the second phase of the formalin test.	N/A	Abad et al. (2011) [125]
*Acorus calamus* extract 100 and 200 mg/kg bworally	Wistar rats	Reversed pain sensitivity in hot plate, plantar, Randall–Selitto, and von Frey hair tests.	⇓ TCA ⇓ Superoxide anion ⇓ MPO	Muthuraman et al. (2011) [132]
*Matricaria chamomilla* extract 25 mg/kg bw i.p.	NMRI mice	Significantly decreased pain responses in both phases of the formalin test.	N/A	Nouri et al. (2012) [126]
Ginkgo biloba extract 50, 100, and 150 mg/kg bw p.o.	Sprague Dawley rats	Significantly reduced mechanical and thermal sensitivity in von Frey, hot plate, and acetone tests in a dose-dependent manner.	N/A	Park et al. (2012) [127]
*Butea* *monosperma* extract 200, 300 and 400 mg/kg bw orally	Wistar rats	Attenuated pain behavior in hot plate, Randall–Selitto, and tail immersion tests.	⇑ GSH ⇓ MDA ⇓ TCA	Thiagarajan et al. (2013) [133]
Gastrodin 0.05–0.8 mg/kg bw i.p.	C57BL/6 mice	Reduced mechanical hyperalgesia in the von Frey test in a dose-dependent manner.	N/A	Guo et al. (2013) [139]
*Vernonia cinerea* extract 200, 300, and 400 mg/kg bw orally	Wistar rats	Significantly reversed thermal hyperalgesia in the hot plate and tail immersion tests, mechanical hyperalgesia in Randall–Selitto and von Frey tests, and thermal allodynia in an acetone drop test.	⇑ GSH ⇓ TBARS ⇓ TCA	Thiagarajan et al. (2014) [134]
*Xylopia aethiopica* Ethanolic extract 30–300 mg/kg bw *OR* Diterpene xylopic acid 10–100 mg/kg bw orally	Sprague Dawley rats	Exhibited hyperalgesia, tactile, and cold anti-allodynic properties in von Frey, Randall–Selitto, and cold water tests, with a more pronounced effect for diterpene xylopic acid.	N/A	Woode et al. (2014) [128]
Matrine 13, 30, and 60 mg/kg bw i.p.	Mice	Reduced pain sensitivity in von Frey cold plate and plantar tests.	N/A	Linglu et al. (2014) [144]
*Ocimum sanctum* Hydro-alcoholic extract *OR* Saponin fraction 100–200 mg/kg bw orally	Wistar rats	Attenuated pain sensitivity in acetone, hot plate, pinprick, and tail immersion tests.	⇓ TBARS ⇓ TCA ⇓ Superoxide anion	Kaur et al. (2014) [135]
*Palisota hirsuta* extract 30–300 mg/kg bw orally	Sprague Dawley rats	Significantly ameliorated mechanical hyperalgesia, thermal hyperalgesia, and cold and tactile allodynia in Randall–Selitto, Hargreaves, cold water, and von Frey tests in a dose-dependent manner.	N/A	Boakye-Gyasi et al. (2014) [129]
Curcumin 15, 30, and 60 mg/kg bw orally	Swiss albino mice	The higher dose reversed thermal (hot–cold plate tests), mechanical sensitivity (pinprick test), functional loss (rotarod test), and reduced pain in the delayed phase of the formalin test.	⇑ SOD ⇑ CAT ⇑ GPx ⇑ GSH ⇓ NO ⇓ LPO ⇓ TCA	Babu et al. (2015) [136]
*Synedrella nodiflora* extract 100, 300, and 1000 mgorally	Sprague-Dawley rats	Attenuated mechanical and thermal hypersensitivity in von Frey, hot plate, tail immersion, and Randall–Selitto tests.	N/A	Amoateng et al. (2015) [130]
Tetrahydrocurcumin 40 and 80 mg/kg bw p.o.	Wistar rats	Higher dose attenuated pain sensitivity in hot–cold plate, Randall–Selitto, and formalin tests.	⇑ MNCV ⇑ SOD ⇑ CAT ⇑ GPx ⇑ GSH ⇓ LPO ⇓ NO ⇓ TNF-α ⇓ TCA	Greeshma et al. (2015) [146]
Fucoidan 50, 100, or 200 mg/kg bw i.p.	Sprague Dawley rats	Repeated administration reduced mechanical and thermal sensitivity in von Frey and acetone tests.	-upregulated GABA-B receptor expression	Hu et al. (2016) [149]
Matrine 15, 30, and 60 mg/kg bw i.p.	Mice	Attenuated mechanical (paw pressure and von Frey tests) and thermal sensitivity (hot–cold plate test) in a dose-dependent manner.	⇑ T-AOC ⇑ IL-10 ⇑ GPx ⇑ SOD ⇑ TCA ⇓ MPO ⇓ MDA ⇓ TNF-α ⇓ IL-6	Gong et al. (2016) [145]
*Tithonia tubaeformis* extract 100 and 200 mg/kg orally	Balb/c mice	Reduced mechanical allodynia and thermal hyperalgesia in von Frey and tail immersion tests.	N/A	Nawaz et al. (2018) [131]
*Tribulus terrestris* saponins 25, 50, and 100 mg/kg bw orally	Wistar rats	Significantly reduced pain sensitivity in Randall–Selitto, von Frey, and formalin tests.	⇑ SNCV ⇓ TNF-α ⇓ IL-1β ⇓ IL-6 ⇓ L-glutamic acid ⇓ L-aspartic acid	Gautam et al. (2019) [137]
Morin 25, 50, and 100 mg/kg bw p.o.	Sprague Dawley rats	Reduced pain hypersensitivity in von Frey and radiant heat source tests.	⇑ SNCV ⇓ IL-6 ⇓ NF-κB ⇓ pNF-κB	Jiang et al. (2019) [150]
Bergapten 10 mg/kg bw i.p.	Wistar rats	Alleviated pain sensitivity in pressure application measurement, von Frey, and tail immersion tests.	⇑ GSH ⇓ TNFα ⇓ IL-1β ⇓ NF-kB ⇓ COX-2 ⇓ iNOS ⇓ MDA	Singh et al. (2019) [151]
Levo-corydalmine 5, 10, and 20 mg/kg bw p.o.	ICR mice	Attenuated thermal and mechanical sensitivity in tail-flick and von Frey tests	⇑HO-1/CO expression ⇑ Nrf2 ⇓ Cx43	Zhou et al. (2020) [153]
Puerarin 25 and 50 mg/kg bw orally	Sprague Dawley rats	Reduced thermal and mechanical sensitivity in hot plate and von Frey tests.	⇑ TGF-β ⇑ IL-10 ⇑ p-Smad2 ⇑ p-Smad3 ⇓ TNF-α ⇓ IL-1β ⇓ NF-κBp65	Xie et al. (2020) [159]
Withametelin 1 mg/kg bw i.p.	Balb/c mice	Reduced thermal (hot plate and acetone tests) and mechanical (von Frey test) hypersensitivity.	⇑ Bcl-2 ⇑ GSH ⇑ GST ⇑ CAT ⇑ SOD ⇓ MPO ⇓ MDA ⇓ NO ⇓ TRPV1 expression levels ⇓ TRPM8 expression levels ⇓ P2Y nociceptors ⇓ ERK ⇓ JNK ⇓ p-38 ⇓ IL-1β ⇓ TNF-α ⇓ Bax ⇓ Casp-3	Khan et al. (2021) [162]
Gastrodin 60 mg/kg bw i.p.	Sprague Dawley rats	Alleviated mechanical and thermal hyperalgesia in von Frey and thermal radiation meter tests.	⇓ NaV1.7 over-expression ⇓ NaV1.8 over-expression	Wang et al. (2021) [140]
Gastrodin 60 and 120 mg/kg bw i.p.	Sprague Dawley rats	Reduced mechanical and thermal hypersensitivity in von Frey and Tottenham pain instrument tests.	⇓ Iba-1 ⇓ CX3CL1 ⇓ TNF-α ⇓ IL-1β ⇓ P-P38	Qin et al. (2021) [141]
Thymoquinone 2.5, 5, and 10 mg/kg bw orally	Albino mice	Reduced pain sensitivity in hot plate, cold plate, and formalin tests and fall-off time in the rotarod test.	⇑ GSH ⇓ MDA ⇓ IL-6	Alenezi et al. (2022) [172]
5,7-Dimethoxycoumarin 30, 40, and 50 mg/kg bw i.p.	Balb/c mice	Reduced pain sensitivity in tail immersion, cotton bud, acetone and von Frey tests.	⇑inosine levels ⇑ vitamin C ⇑ adenosine levels ⇓ serotonin levels ⇑ noradre-naline levels ⇓ dopamine levels ⇓ TNF-α	Usman et al. (2023) [152]
L-theanine 30, 100, and 300 mg/kg bw i.p.	Wistar rats	Reduced thermal hyperalgesia and allodynia in hot plate and acetone tests and mechanical hyperalgesia and allodynia in paw pressure and von Frey tests.	⇑ MNCV ⇑ SNCV ⇑ GSH ⇑ SOD ⇑ CAT ⇑ IL-10 ⇓ NO ⇓ MDA ⇓ Casp-3 ⇓ TNF-α ⇓ IL-6 ⇓ MPO	Yang et al. (2023) [173]

⇑-increased; ⇓-decreased; Bax-Bcl2—associated X-protein; Bcl-2—B-cell-lymphoma-2; bw—body weight; Casp3—caspase 3; CAT—catalase; CO—carbon monoxide; COX2—cicclooxygenase 2; CX3CL1—fractalkine; CX43—connexin 43; ERK—extracellular signal-regulated kinase; GPx—glutathione peroxidase; GSH—reduced glutathione; GST—glutathione-S-transferases; HO-1—heme oxygenase 1; i.p.—intraperitoneally; i.t.—intrathecally; Iba-1—ionized calcium-binding adaptor molecule 1; IL-10—interleukin 10; IL-1β—interleukin 1β; IL-6—interleukin 6; iNOS—inducible nitric oxide synthase; JNK—c-Jun N-terminal kinase; LPO—lipid peroxidation; MDA—malondialdehyde; MNCV—motor nerve conduction velocity; MPO—myeloperoxidase; N/A-not applicable; NADPH—nicotinamide adenine dinucleotide phosphate; NFL—neurofilament light chain; NF-κB—transcription factor nuclear factor kappa B; NO—nitric oxide; Nrf2—NF-E2 related factor-2; P-P38—phosphorylated form of the p38 protein; pNF-κB—phosphorylated NF-κB; p-Smad2—phosphorylated Smad 2 (mothers against decapentaplegic homolog 2); p-Smad3—phosphorylated Smad 3 (mothers against decapentaplegic homolog 3); ROS—reactive species of oxygen; SNCV—sciatic nerve conduction velocity; SOD—superoxide dismutase; T-AOC—total antioxidant capacity; TBARS—thiobarbituric acid reactive species; TCA—total calcium; TGF-β—transforming growth factor ß; TNF-α—tumor necrosis factor α; TRPM8—Transient receptor potential melastatin 8; TRPV1—transient receptor potential vanilloid.

**Table 3:** Preclinical studies investigating the effects of antioxidants and vitamins on VIPN.

Tested Substances/Doses	Animals	Results	Involved Mechanism	Reference
Vitamin E 50, 100, and 200 mg/kg bw orally	Wistar rats	Higher doses attenuated pain sensitivity in the tail flick, tail immersion, and tail clip tests.	N/A	Nagkrishna et al. (2013) [184]
Thioctic acid 1, 5, and 10 mg/kg bw i.p.	Sprague Dawley rats	Reduced mechanical and cold allodynia in von Frey and acetone tests.	N/A	Kahng et al. (2015) [175]
Methylcobalamin 0.12, 0.5, and 1 mg/kg bw i.p.	Sprague Dawley rats	Reduced mechanical allodynia (von Frey test) and thermal hyperalgesia (plantar test).	-inhibited the loss of IENF and prevented mitochondria impairment ⇑ IL-10 ⇓ NADPH oxidase ⇓ p-p65 ⇓ TNF-α	Xu et al. (2016) [186]
Ferulic acid 50 and 100 mg/kg bw i.p.	Wistar rats	Reduced mechanical and heat hyperalgesia in pinprick and hot plate tests and mechanical and cold allodynia in von Frey and acetone tests.	⇑ GSH ⇑ IL-10 ⇓ TCA ⇓ MPO ⇓ TNF-α ⇓ IL-6 ⇓ TBARS	Vashistha et al. (2017) [177]
Mitoquinone 2.5, 5, and 10 mg/kg bw i.p.	ICR mice	Improved pain sensitivity in von Frey and tail-flick latency tests.	⇑ SOD ⇑ GSH ⇑ Nrf2 ⇑ Bcl-2 ⇓ ROS ⇓ H_2_O_2_ ⇓ NADPH oxidase activity ⇓ MDA ⇓ IL-6 ⇓ COX2 ⇓ IL-18 ⇓ IL-1β ⇓ TNF-α ⇓ Iba1 ⇓ GFAP ⇓ CX3CR1 ⇓ CCR2 ⇓ Casp 3 ⇓ Bax ⇓ Cyto-C release	Chen et al. (2020) [187]
Coenzyme Q10 10 mg/kg bw i.p.	Sprague Dawley rats	Attenuated thermal hypersensitivity in tail immersion and acetone tests.	⇑ Nrf2 mRNA gene expression ⇑ TAC ⇑ GSH ⇓ MDA ⇓ 8-OHdG ⇓ TNF-α ⇓ IL-1b ⇓ NF-κB ⇓ NFL ⇓ SARM1 mRNA gene expression	Elshamy et al. (2022) [181]

⇑-increased; ⇓-decreased; 8-OhdG—8-Hydroxy-2′-deoxyguanosine; bw-body weight; Bax-Bcl2—associated X-protein; Bcl-2—B-cell-lymphoma-2; Casp 3—Caspase 3; CCR2—C-C motif chemokine receptor 2; COX2—cicclooxygenase 2; CX3CR1—C-X3-C motif chemokine receptor 1; Cyto-C—cytochrome C ; GFAP—glial fibrillary acidic protein; GSH—reduced glutathione; Iba1—Ionized Calcium-Binding Adapter Molecule 1; IENF—intraepidermal nerve fiber; i.p.—intraperitoneally; IL-10—interleukin 10; IL-1β—interleukin 1β; IL-6—interleukin 6; IL-18—interleukin 18; N/A-not applicable; MDA—malondialdehyde; MPO—myeloperoxidase; NADPH—nicotinamide adenine dinucleotide phosphate; NFL—neurofilament light chain; Nrf2—NF-E2 related factor-2; p-p65—phosphorylated p65, ROS—eactive species of oxigen; SARM1—sterile alpha and TIR motif containing 1; SOD—superoxide dismutase; TAC—total antioxidant capacity; TBARS—thiobarbituric acid reactive species; TCA—total calcium; TNF-α—tumor necrosis factor α.

## Data Availability

All data generated or analyzed during this study are included in this published article.
